# Role of ncRNAs in Hepatocellular Carcinoma

**DOI:** 10.1155/2018/3014543

**Published:** 2018-07-31

**Authors:** Kangsheng Tu, Qiongzhu Dong, Junyan Tao

**Affiliations:** ^1^Department of Hepatobiliary Surgery, The First Affiliated Hospital of Xi'an Jiaotong University, Xi'an, China; ^2^Institutes of Biomedical Sciences, Fudan University, Shanghai, China; ^3^School of Medicine, University of Pittsburgh Medical Center, Pittsburgh, USA

Hepatocellular carcinoma (HCC) is one of the most common malignant tumors worldwide and a major cause of cancer-related mortality in China. Most cases of HCC are diagnosed in the advanced stage. Until now, Sorafenib and Regorafenib, two FDA approved drugs, only can prolong 2-3-month survival for unresectable HCC patients. So the advance on molecular mechanisms under HCC progress might sled on its diagnosis and treatment. Increasing studies figure out that noncoding RNAs (ncRNAs), a class of RNAs with no protein-coding function that are widely expressed in tumor, play an important role in tumor progression including HCC. This special issue provided a platform to introduce and disclose the role of ncRNAs in HCC. At last, five articles, including three review articles and two research articles, were accepted for publication. Here, three guest editors summarily introduced these published articles in this editorial.

J. Yang et al. explored the role of miR-219-5p in tumor growth and metastasis of human HCC. They revealed that elevated expression of miR-219-5p was positively correlated with poor prognostic features including liver cirrhosis, vascular invasion, and poor differentiation. Moreover, miR-219-5p upregulation was recognized as an independent predictor for dismal prognosis of HCC patients. Functionally, miR-219-5p contributed to cell proliferation and invasion of HCC cells* in vitro* and* in vivo* by targeting cadherin 1 (CDH1). The regulatory mechanism underlying aberrant expression of miR-219-5p and the novel targets involved in the oncogenic role of miR-219-5p in HCC should be further investigated.

Accumulating studies verify that liver cancer stem cells (CSCs) account for tumor initiation, metastasis, recurrence, and chemoresistance. J. Zhao et al. summarized the recent literatures regarding the diverse regulatory role of two main classes of ncRNAs, miRNAs, and lncRNAs, in CSC maintenance. They reviewed miRNAs and lncRNAs regulation of signaling pathways, stemness-related transcriptional factors and markers, tumor-associated genes,* etc.* in the maintenance of liver CSCs. The critical roles of miRNAs and lncRNAs in the regulation of liver CSCs will provide new idea to develop therapeutic strategies for liver cancer by targeting ncRNAs.

H. Zhou et al. reported a review on recent knowledges about the ncRNAs in the development of HCC. Notably, abnormally expressed microRNAs in tissues and serum of patients may serve as prognostic or diagnostic biomarkers of HCC. Furthermore, ncRNAs may be involved in a variety of pathological processes such as cell proliferation, apoptosis, angiogenesis, invasion, and metastasis. In addition to lncRNA and miRNA, other ncRNAs, including circRNA and snoRNA, also may have significant influence on the development and progression of HCC.

To identify ncRNAs involved in HCC, T. Falcon et al. analyzed total RNA from 41 pairs of primary solid tumor and adjacent tissue samples from The Cancer Genome Atlas (TCGA) using the function GDCdownload with the option legacy = FALSE. They found that there are 234 miRNAs, 92 pre-miRNAs, and 122 lncRNAs differential expression between tumor and normal samples. Then the authors explored the pathways that differentiate both groups and the regulatory ncRNAs and their putative targets. They found that the most represented pathways in differentially expressed transcripts are involved in bile metabolism, fear behavioral response, and immune-related categories. A set of lncRNAs includes FAM170B-AS1 and TTNAS1 that could be the potential targets of future studies in HCC. These two papers indicated that ncRNAs may be promising prognostic biomarkers of HCC and therapeutic targeting of ncRNAs may be a potential strategy for preventing progression in patients with HCC.

X. Hu et al. gave a system landscape on the role of LncRNAs in HCC development based on basic research and clinic study, including various tumor suppressor and oncogenic lncRNA, the signal pathway involved in lncRNA deregulation, and function in HCC development. Meanwhile, they also discussed the potential role of lncRNA for HCC diagnosis, recurrence, and outcome prediction and target therapy. Based on the review on lncRNA and HCC, it will help us to expand our current research horizon, to promote the diagnosis and treatment study on HCC in further.

*Kangsheng Tu* *Qiongzhu Dong* *Junyan Tao*

